# Ceftriaxone Administration Associated with Lithiasis in Children: Guilty or Not? A Systematic Review

**DOI:** 10.3390/jpm13040671

**Published:** 2023-04-16

**Authors:** Aspasia Louta, Aimilia Kanellopoulou, Loukia Alexopoulou Prounia, Mathiou Filippas, Faidra Foteini Tsami, Athanasios Vlachodimitropoulos, Antonios Vezakis, Andreas Polydorou, Ioannis Georgopoulos, Despoina Gkentzi, Ioannis Spyridakis, Ageliki Karatza, Xenophon Sinopidis

**Affiliations:** 1 Second Department of Surgery—Intensive Care Unit and Endoscopy Unit, Aretaieion University Hospital, 11528 Athens, Greece; 2 School of Medicine, University of Patras, 26504 Patras, Greece; 3 Third Department of Psychiatry, Dromokaition Psychiatric Hospital, 12461 Athens, Greece; 4 Endoscopy Unit, Second Department of Surgery, Aretaieion Hospital, National and Kapodistrian University of Athens, 11528 Athens, Greece; 5 Surgical Department, ‘Agia Sofia’ Children’s Hospital, 11527 Athens, Greece; 6 Department of Pediatrics, University General Hospital of Patras, University of Patras School of Medicine, 26504 Patras, Greece; 7 Second Department of Pediatric Surgery, Aristotle University of Thessaloniki, Papageorgiou General Hospital, 56429 Pavlos Melas, Greece; 8 Department of Pediatric Surgery, University General Hospital of Patras, University of Patras School of Medicine, 26504 Patras, Greece

**Keywords:** ceftriaxone, children, lithiasis, pseudocholelithiasis, stones, urolithiasis, pediatric patients, nephrolithiasis

## Abstract

Lithiasis is a known side effect of ceftriaxone administration in children. Sex, age, weight, dosage, and duration of intake have been reported as risk factors for the formation of calcification or stones in the bile and urine excretory systems of children who received ceftriaxone. The purpose of this systematic review is to investigate the reported effects of ceftriaxone administration in pediatric patients who were admitted to a hospital due to infection, the likelihood of gallstones, nephroliths, or precipitations in both the biliary and urinary systems, as well as investigate the relationship with their mother’s history during pregnancy. Original studies and literature reviews from the PubMed database were included in the study. No time limit related to research or publication was set for the articles. The results were evaluated, aiming to understand the outcomes and identify any predisposing factors relevant to this side effect. Of the 181 found articles, 33 were appropriate for inclusion in the systematic review. The administered dose of ceftriaxone presented variability. Symptoms, such as abdominal pain and vomiting, were associated with ceftriaxone-related lithiasis in many cases. It was noted that most of the results were the outcomes of retrospective observation and not of prospective randomized research. Definitively, more randomized control studies with long-term outcomes are needed to identify the exact association between ceftriaxone and lithiasis in children.

## 1. Introduction

Ceftriaxone, introduced in 1984, is a third-generation cephalosporin that is effective against a broad range of micro-organisms with favorable tissue penetration and is widely used for a variety of serious infections [1]. It is administered parenterally, binds to serum proteins, and has a long plasma half-life, often permitting a single daily dose. It exhibits minimal dose-dependent pharmacokinetics since its free fraction in the serum is concentration-dependent [2]. It is mainly excreted via urine, but a significant amount is secreted (unmetabolized) through the biliary system [3].

Biliary pseudolithiasis is a well-documented side effect of ceftriaxone, first described in 1986 [4]. It occurs in 15–46% of children treated with ceftriaxone, and it is resolved after interruption of administration, justifying the term pseudolithiasis [5,6]. The high calcium-binding affinity of ceftriaxone has been suggested as the main pathogenetic factor of precipitation and formation of calcification and stones in the biliary tracts [7]. Occasionally, ceftriaxone-induced pseudolithiasis may cause severe complications, such as obstruction and infection [8,9]. Therefore, more interventions for the resolution of biliary lithiasis have occasionally been applied, such as the use of ursodeoxycholic acid [10]. The predisposing factors for calcification or gallstone formation have been investigated according to the characteristics of children with biliary pseudolithiasis, such as age, gender, body weight, dose, fasting, and bed rest [6,11]. The method of administration, i.e., bolus injection versus 30 min of drip infusion, and its role in the occurrence of cholelithiasis has also been studied [12]. Prenatal diagnosis of ceftriaxone-associated cholelithiasis has been reported, even in pregnancy [13].

Apart from biliary pseudolithiasis, a strong association between nephrolithiasis and ceftriaxone therapy in children has been reported, with an incidence of 1.4% [14,15]. This type of nephrolithiasis may rarely evolve into acute urinary retention, acute renal injury, and hydronephrosis [16]. Although ceftriaxone-related urolithiasis or calcification in the urinary tract is believed to be rare, the increased use of ultrasonography has led to increased detection rates [17]. Urine pH seems to play a role in the increased urinary concentration of calcium and is related to ceftriaxone-associated calcification and lithiasis [17,18]. Obstruction, infection, genetic polymorphisms, immunodeficiencies, hemolytic anemia, and anomalies of the urogenital tract may favor nephrolithiasis [19,20]. Ceftriaxone-related nephrolithiasis has been associated with alterations to the intestinal microbiome [21]. Finally, duration of therapy is a known risk factor for both biliary pseudolithiasis and nephrolithiasis [22,23].

The aim of this systematic review is to investigate what is known and what is new regarding the association between ceftriaxone and biliary and urinary tract lithiasis in children and to outline possible prevention strategies.

## 2. Methods

We performed a literature search with the following inclusion criteria for article types: original articles, reviews, meta-analyses, case reports, articles obtained in full text, articles in English, and articles on humans. No time limit was set for articles related to their research or publication.

The database used was PubMed/Medline. The keyword combinations were ceftriaxone, children, and lithiasis (*n* = 20 articles), ceftriaxone, children, and pseudocholelithiasis (*n* = 4), ceftriaxone, children, and stones (*n* = 56), ceftriaxone, children, and urolithiasis (*n* = 27), ceftriaxone, pediatric patients, and stones (*n* = 18), ceftriaxone, pediatric patients, and lithiasis (*n* = 7), ceftriaxone, children, and urolithiasis (*n* = 27), ceftriaxone, children, and nephrolithiasis (*n* = 22). In total, 181 articles were extracted. The principles of the preferred reporting items for systematic reviews and meta-analyses (PRISMA) methodology were used for the analysis of the retrieved information [24]. The review has been registered in the international prospective register of systematic reviews.

## 3. Results

**Figure 1:**
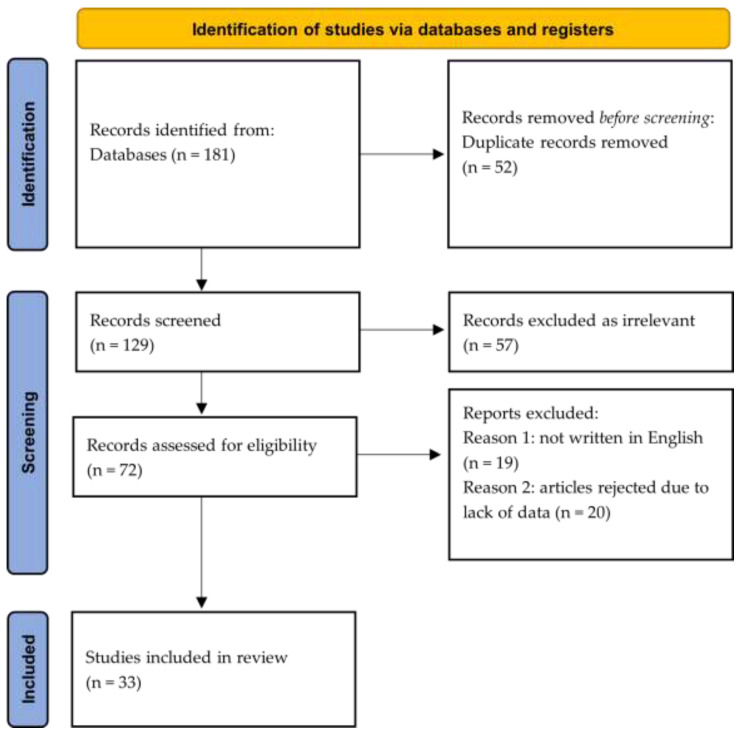
Flow diagram of literature search and article selection process according to the PRISMA guidelines [24].

Of the 181 articles, 52 were not included because their title and content did not refer to the development of lithiasis or pseudolithiasis. Of the remaining 129 articles, some reappeared multiple times and, therefore, were removed, resulting in a total of 72. Of the remaining articles, 19 were not written in English and were excluded. Eventually, 53 articles were considered suitable for inclusion in the systematic review; however, 20 of these were eliminated due to their lack of relevant data and were, therefore, rejected (Figure 1). Eventually, 33 studies were included. Of those, 12 were case reports, 11 were prospective studies, nine were retrospective studies, and only one was a randomized controlled trial. Twenty-one studies reported evidence of biliary calcification, biliary pseudolithiasis, or gallstones in patients who had received or were receiving ceftriaxone during the time of the detection of lithiasis. Seven studies regarded lithiasis of the urinary tract, and five studies presented data combining both systems. The outcomes for ceftriaxone administration and the clinical presentation of lithiasis, as well as the clinical and laboratory findings and outcomes, are outlined for biliary lithiasis (Table 1), urinary lithiasis (Table 2), and combined biliary and urinary lithiasis presentation (Table 3).

## 4. Discussion

A systematic review was performed to assess the association between ceftriaxone administration and lithiasis in children. The results of the identified studies were analyzed, aiming to understand the side effects and detect any predisposing factors. In this section, the clinical and laboratory expressions of crystal formation regarding distinct biliary, urinary, and combined lithiasis are discussed in terms of pathophysiology, clinical presentation, diagnosis, and outcomes.

### 4.1. Biliary Tract Lithiasis

Ceftriaxone is the most commonly associated risk factor for pediatric cholelithiasis. The incidence of ceftriaxone-associated cholelithiasis has been defined as 15–46% [5,27]. In a large retrospective study, it was associated with 20% of all children with crystal formation [20]. The drug was considered a lithiasis risk factor within the infancy age group [20].

Ceftriaxone is excreted unmetabolized into the bile, resulting in a concentration 20–150 times greater than in the serum [7]. The relocation of the anion-charged drug through hepatocytes is mediated by transporting peptides [47]. Furthermore, its high calcium-binding affinity results in the formation of insoluble salts with calcium deposited into the bile. The biochemical process of pH-influenced binding of ceftriaxone with calcium and salt formation [18] is of clinical interest. The aggregation of ceftriaxone-calcium salts into the bile may result in sedimentation and the formation of calcification or even lithiasis.

Palanduz et al. considered age not to be associated with biliary lithiasis [35]. Gender was considered irrelevant with lithiasis as well [26,28,35]. The mean age of the patients reported in the case reports with lithiasis of the biliary tract in this study was 6 years (Table 1).

Lithiasis was associated with excessive daily doses [6,18]. The majority of studies reported a high dose of at least 100 mg/kg/d [11,12,23,28,30,31,34,35,38]. Four studies reported a daily dose of 50–100 mg/kg [18,34,35,36]. Pseudolithiasis occurred even in daily doses under 60 mg/kg/d [35] (Table 1).

The administration period until the diagnosis of lithiasis was under 10 days [25,28,37] and from 1 to 3 weeks [30,35,38]. In one study, it was 50 days [23]. Duration of administration was not associated with pseudolithiasis by all researchers [25]. A finding of interest from the only randomized controlled study was that the combination of an age of over 12 months, a daily dose of more than 2 g, and a duration of therapy longer than 5 days were associated with biliary precipitations [37]. Bacterial meningitis was associated with a higher presentation of gallstones, requiring higher doses and a prolonged treatment period, which reached even 60 days [12] (Table 1).

Abdominal pain, fever, a positive Murphy’s sign, and vomiting frequently occurred between the fourth and seventh day after administration [30,32,33,43]. Abdominal pain was reported to initiate even after the discontinuation of ceftriaxone [38].

Tuna Kirsaclioglu et al. observed that ceftriaxone could lead to stable gallstones and complicating diseases, such as cholecystitis [20]. Fasting (with lithiasis reported in the first week of administration), bed rest (lithiasis reported during the first 5 days), feeding habits, and activity patterns are the factors considered to affect lithiasis [11,36]. A family history of lithiasis has been reported as an important risk factor for the formation of gallstones [36]. Although ceftriaxone is often used with other drugs, it has been exclusively associated with lithiasis [23,26,33] (Table 1).

The detection time of lithiasis using ultrasound is reported to be between 10–20 days [12,25,27]. However, there are studies that report a diagnosis time of less than a week [26,31,32,34,35,37]. Cholelithiasis was detected even after 35 h [18]. Biliary calcification and gallstones were the main findings using ultrasonography [29,48] (Table 1).

Bor et al. determined that ceftriaxone-related cholelithiasis was most likely to dissolve in up to a maximum period of 90 days [27]. Most cases of pseudolithiasis were resolved during the first week after ceftriaxone discontinuation [26,27,28,43,45]. Resolution occurred after up to 3 months as well [6,23]. Lithiasis persisted in one patient for 7 months [12]. It was reported that patients whose gallstones dissolved were significantly younger (mean age: 8 years) compared to other patients with cholelithiasis of different etiology (mean age: 9.5 years) [20].

### 4.2. Urinary Tract Lithiasis

Nephrolithiasis and crystalline nephropathy are induced by numerous drugs, with 1–2% of all urinary stones occurring due to drug administration. Two pathogenetic mechanisms have been described. The first includes poorly soluble drugs with high urine excretion, which favors crystallization [49]. Antiseptic molecules, such as sulfonamides, the first historically implicated drugs in the formation of renal calculi [50], and ceftriaxone are included in this process, and their molecules have been identified in the crystals. The drugs that activate the formation of urinary calculi as a metabolic effect on urinary pH or the excretion of calcium, phosphate, oxalate, citrate, uric acid, or other purines are included in the second mechanism [51]. In this case, the drug molecules are not contained in the crystals, rendering their identification difficult in terms of causative factors [52].

Kimata et al. demonstrated that ceftriaxone could significantly increase the urinary excretion of calcium in children, inferring that this mechanism may be associated with lithiasis or calcification formation [17]. Transient hypercalciuria and the elevated excretion of oxalic and uric acid have been identified as well [53]. Nevertheless, there are studies on crystal formation with normal urine calcium or other salt levels [22,46] (Table 2 and Table 3).

The formation of urine crystals after ceftriaxone administration is affected by a variety of risk factors, such as urinary stasis, family history, metabolic abnormalities (i.e., hypercalciuria), abnormal urine pH, infection, environmental factors (i.e., high temperature), low urine output, high drug dosage, long treatment, high urinary drug excretion, and concomitant therapies [48,54].

Urinary tract lithiasis presents in a wide age range from infancy to puberty. Age has been considered as a possible associated factor [6,22]. Half of the children with urinary lithiasis included in Table 2 were under the age of 3 years, while four children were younger than one year (Table 2). In their prospective study, Fesharakinia et al. correlated male gender with nephrolithiasis [44]. It is of interest that only one study reported a low dose of 50 mg/kg/d, which was combined with hypercalciuria [40]. Doses of 100 mg/kg/d [19,44] and 50–100 mg/kg/d [17,22] were reported in patients with urolithiasis. In two studies, administration lasted less than 10 days [22,42]. Shen et al. treated patients with acute kidney injuries induced by ceftriaxone, which had been referred to after ceftriaxone had been administered in extremely high doses. In these overdosed patients, cystoscopy and ureteral catheterization were performed. All patients recovered except for one, who underwent surgical excision for the nephrolith [42].

The detection of lithiasis occurred within 10–20 days [18,41]. Kidney stone formation was the most common finding. Urinary bladder calcification subsided when ceftriaxone was discontinued [43]. Smaller calculi were excreted more easily in the first weeks after the discontinuation of administration, while larger stones lasted for months [22]. Although resolution occurred early (until the end of the first week) [29,43], there were cases of resolution that occurred after 7 months [39]. In a study with a follow-up period of 3 years, no recurrent urolithiasis was reported [42].

### 4.3. Combined Biliary and Urinary Tract Lithiasis

Lithiasis of both excretory systems was reported in patients from 1 month to 18 years in the articles of this study (Table 3). Two prospective studies reported an association with age, particularly in patients that were older than 5 years [6,46]. Fesharakinia et al. reported a presentation of 6.3% urolithiasis and 1% cholelithiasis [44].

Acun et al. attributed the lithiasis of the biliary and urinary bladder calculi to a combination of high dosage (100 kg/d) and low infusion flow (20 min) [43]. High doses of 100 mg/kg/d and patients with bacterial meningitis were considered risk factors [6]. As in the Acun et al. study, in which the administration time was short, in the Biner et al. study, the drug was administered in bolus doses [6,43]. The doses implicated in this group were 100 mg/kg/d [43,45] and 50–100 mg/kg/d [6,44] (Table 3). The duration of ceftriaxone administration lasted for 10 days [6] and from 1 to 3 days [44,46] until lithiasis was diagnosed. There was no association between lithiasis and the season of hospitalization [44].

Ustyol et al. reported behavior that was analogous to the ceftriaxone behavior of cefotaxime in a third prospective study, with 40% of the drug binding to serum proteins and 60% being excreted through the urinary tract [46], inferring a wider third-generation cephalosporin association with lithiasis. However, ceftriaxone-associated lithiasis prevailed over that of cefotaxime.

Lithiasis resolution occurred in all the articles of this study. In the prospective study of Biner et al., in which 17% of patients who received ceftriaxone presented biliary and 0.6% urinary lithiasis, all the patients recovered completely after the discontinuation of the antibiotic [6]. In all patients who co-operated with the follow-up of the research, no crystals were identified after the discontinuation of treatment [44].

### 4.4. Limitations

This review has certain limitations. Although being the most thoroughly used in medicine, the database that we used does not completely cover all available information. With the inclusion of articles only written in the English language, we may have excluded some interesting articles in other languages. Most of the articles included in the final database were of a retrospective nature or case reports, implying consistency biases in data collection and validation.

## 5. Conclusions

Although any form of ceftriaxone-related lithiasis consists of one of the known etiologies of pediatric stone formation, the final number of research articles justifying the inclusion criteria was small; most importantly, they included only one randomized controlled study. Thus, the information recorded was a result of observation rather than scientific documentation at a higher level. Definitively, more randomized control studies with long-term outcomes are needed.

It is of note that the clinical presentation and diagnosis of lithiasis are related to the dose and duration of administration. However, more parameters should be questioned as potential risk factors, such as dietary habits, fasting, and bed resting, especially when an antibiotic is mandatory due to severe infectious diseases, such as bacterial meningitis, or longstanding conditions, such as bed rest in hospitals. It is noteworthy that a combination of many factors apart from the dose of the antibiotic may contribute to a lithiasis outcome.

As a clinical implication in the future for the prevention and management of ceftriaxone-induced lithiasis, the clinician should anticipate crystal formation in the biliary and urinary systems when ceftriaxone is administered, especially in high doses with a fast administration rate and for a prolonged time. There should be an intense focus on clinical signs and serum and urine calcium levels, as well as routine liver and urine tests and renal ultrasounds should be performed at the end of the first week from the initiation of ceftriaxone administration. Thus, if crystal formation occurs, the discontinuation of an antibiotic may result in resolution because of a timely reaction.

In order to provide an answer to the title of the study, the drug is not guilty. Under wise usage and thorough consideration of its possible side effects, crystal formation may be anticipated and prevented. It is a valuable tool in the hands of the clinician, who must use it with prudence, taking into consideration the presence of the predisposing lithiasis parameters and taking timely measures for the screening and diagnosis of the side effects.

## Figures and Tables

**Table 1:** Summary of the main findings from the articles included in the study for exclusively biliary ceftriaxone-related complications.

Citation	Study Type	Study Patients (*n*)	Age, Gender	Clinical Information, Treatment	Main Outcomes
Alemayehu et al. [25]	Retrospective	*n* = 71 Stones or calcification in the gallbladder were found in *n* = 10 (14%) patients. mean age 10.0 ± 4.9 y.	Mean age: 10.8 ± 3.8 yr Gender undefined	Ceftriaxone 50 mg/kg, max. dose 2 g, infusion over 10 min. Mean duration of treatment: 8.7 ± 3.8 d. Postoperative administration for laparoscopic appendectomy.	Ultrasound on 11.5 ± 10.3 d of treatment showed biliary lithiasis. Nonsignificant difference in duration of ceftriaxone treatment between patients who presented gallbladder lithiasis compared to those that did not. Duration of administration was considered not important for lithiasis. Lack of uniformity in the timing of repeat imaging after initiation of treatment. Resolution except for one symptomatic patient who underwent cholecystectomy.
Araz et al. [26]	Case Reports	*n* = 8	Age range: 3–9 yr Mean: 6 ± 2 yr Females: *n* = 1 Males *n* = 7	Ceftriaxone 100 mg/kg/d bolus, *n* = 8 patients. Sultamicillin 100–200 mg/kg/d: *n* = 2 patients. Penicillin-G 100.000 U/kg/d: *n* = 2 patients. Meningitis.	Ultrasound on 7 ± 1 d of treatment. Biliary calcification: *n* = 1. Gallstones: *n* = 3. Biliary calcification and gallstones: *n* = 4. Normal ultrasound in all patients at 41 ± 23 d after discontinuation of ceftriaxone treatment. High summer temperature (*n* = 6 patients) inferred as possible risk factor of lithiasis.
Bor et al. [27]	Prospective	*n* = 38 Patients with lithiasis: *n* = 14 (36.8%). Gender undefined.	Females: *n* = 37 Males: *n* = 13	Ceftriaxone 100 mg/kg/d in two equal bolus doses, infusion time 1–2 min. Meningitis, salmonellosis, gastroenteritis, urinary tract infections, respiratory tract infections, fever of unknown origin.	Abnormal ultrasound after 10 d: *n* = 14 (36.8%). Cholelithiasis *n* = 11 (28.9%) and biliary calcification: *n* = 3 (7.9%). Cholelithiasis after 30 d in patients with abnormal ultrasound: *n* = 2. Cholelithiasis on 60 d: *n* = 1. All cases resolved spontaneously during a maximum period of 90 d.
Ceran et al. [28]	Prospective	*n* = 50 Lithiasis was present in 13 (26%), with an age range of 6.8 ± 4.5 yr.	Age range: 6 mo–16 yr Females: *n* = 14 Males: *n* = 18	Ceftriaxone 100 mg/kg/d in two bolus doses for 3–10 d. Pediatric surgical patients were exposed to starvation because of surgery.	Ultrasonographic biliary pathology: *n* = 13 Resolution of ultrasound findings 7 d after ceftriaxone discontinuation. Duration of administration, age, starvation, and gender were nonsignificant factors for lithiasis.
Dinleyici et al. [12]	Prospective	*n* = 32 Abnormal gallbladder: *n* = 9, 28.1%. Cholelithiasis: *n* = 3, 9.3%. Biliary calcification: *n* = 6, 18.7%.		Ceftriaxone 100 mg/kg/d in 2 doses of drip infusion (30 min). Community-acquired pneumonia, urinary tract infection, meningitis.	Ultrasound at 10 d performed 4–8 h after fasting. Cholelithiasis persisted on ultrasound in male patients of 7 yr with bacterial meningitis (3.1%) and resolved 60 d after therapy. Compared to bolus (older study), drip infusion resulted in significantly less abnormal gallbladder and cholelithiasis.
Dooki et al. [29]	Retrospective	*n* = 66 Cholelithiasis: *n* = 18 (27.3%).	Age range: 2 mo–17 yr Mean: 6.6 ± 4.5 yr Females: *n* = 27 Males: *n* = 39	Study on ultrasound follow-up of cholelithiasis of different etiologies.	Ceftriaxone administration was the most common predisposing factor of pediatric cholelithiasis among a variety of aetiology.
Du et al. [23]	Case report	*n* = 1	Male, 9 yr	Fungal pneumonia and central nervous system infection. Free history of lithiasis prior. Administered ceftriaxone 2 g/d for 50 d, together with fluconazole, ambroxol hydrochloride, and vancomycin. Ursodeoxycholic acid administered 2 wk before hospital discharge.	Hepatic dysfunction, gallbladder sediment, and pathologic biochemical liver tests. CT scan showed reduction in gallbladder volume 5 d after ceftriaxone discontinuation. Gallbladder abnormalities disappeared after 4 mo.
Fretzayas et al. [30]	Case Report	*n* = 3	Female: (*n* = 1), 5.5 mo, Males: (*n (n* = 2), 18 mo, and 4 yr	Ceftriaxone 100 mg/kg/d for 10–14 d. Urinary tract infection. Genetic anomalies.	All patients presented gallbladder pseudolithiasis. Ultrasound findings: intense, mobile, and echogenic material with acoustic shadow. Reduced function of UDP-glucuronosyltransferase encoded by UGT1A1 gene (due to UGT1A1 gene polymorphisms found in all patients) seems to be compatible with pseudolithiasis.
Gokce et al. [10]	Retrospective	*n* = 124 Cephalosporin was administered in *n* = 16 asymptomatic and *n* = 7 symptomatic patients with lithiasis.	Mean age: 9 yr Females: *n* = 63 (Mean: 10.5 yr) Males: *n* = 61 (Mean: 7 yr)	Study on patients with cholelithiasis of various etiologies. Symptomatic lithiasis: *n* = 76 (61%). Asymptomatic lithiasis: *n* = 48 (39%). Ceftriaxone was studied as a possible risk factor.	The risk factors of lithiasis in the symptomatic infant subgroup (*n* = 7) were ceftriaxone use in one (14.3%), prematurity plus ceftriaxone use in one (14.3%), and dehydration plus ceftriaxone use in one (14.3%). In the asymptomatic infant subgroup (*n* = 11), ceftriaxone was administered in 4 (36.4%) patients, as it was considered a risk factor as well.
Ito et al. [18]	Retrospective	*n* = 136 Gallstones presented within 14 d (median 9 d) after administration in 75% of patients in both groups.	Females: *n* = 76 Males: *n* = 57 Nondetermined gender: *n* = 3 Patients under 10 yr: *n* = 53	The study aimed to clarify the factors that favor ceftriaxone and calcium binding and to investigate ceftriaxone-calcium salt formation in vitro. Maximum dose of ceftriaxone: adults: 2 g/d, children: 60 mg/kg/d, which were considered as the upper limits of the normal dose. The study included both children and adults divided into two groups according to age.	Excessive dosage of ceftriaxone was associated with lithiasis. Calcium and pH were factors influencing salt formation. Gender did not affect lithiasis. It is of interest that dose was above normal in 73% of the younger group. Lithiasis occurred more frequently in children compared to adults. Clinical outcomes were tested in vitro.
Krzemien et al. [31]	Case Report	*n* = 1	Male, 5 mo	Ceftriaxone 100 mg/kg/d. Acute pyelonephritis.	After 5 and 11 d of treatment, there was gallbladder enlargement. Therapy continued for 10 d. Gallbladder size became normal after 6 wk.
Kutuya et al. [32]	Case Report	*n* = 1	Male, 5 y	Ceftriaxone 2 g/d in two doses. Pneumonia.	Abdominal pain after 1 wk of treatment. Ultrasound showed a hyperechoic band within a collapsed gallbladder. Therapy was disrupted after diagnosis of lithiasis. At 8 d of treatment, ultrasound showed echoes within the gallbladder neck and the common bile duct and dilatation of the bile duct. At 11 d, there was calcification in the gallbladder. At 13 d, ultrasound findings were normal.
Lemberg et al. [33]	Case Report	*n* = 1	Female, 6 y	Empiric treatment with ceftriaxone and acyclovir. Presumptive meningitis.	Abdominal pain 4 d after hospital admission, with positive Murphy’s sign and high fever. Ultrasound showed a gallstone within the gallbladder. Normal ultrasound findings 3 wk after discharge.
Meng et al. [5]	Prospective	*n* = 108 Ceftriaxone 30–80 mg/kg/d for 1–3 wk in *n* = 58 patients (32 with biliary tract infection and 26 with pneumonia).	Age range: 9 mo–11 yr Mean: 33 mo Females: *n* = 52 Males: *n* = 56	Cephalosporins were administered for biliary tract infection and pneumonia. Ceftazidime 50–100 mg/kg/d for equal period in *n* = 50 patients (29 with biliary tract infection and 21 with pneumonia).	Biliary gallstones and calcification were detected in 25 patients under ceftriaxone and 1 patient under ceftazidime. Gallstone formation was significantly higher in the ceftriaxone group. Resolution of symptoms occurred 1–2 d after discontinuation of treatment. Resolution of ultrasound findings occurred 7–14 d after discontinuation of treatment.
Murata et al. [11]	Retrospective	*n* = 60 Biliary pseudolithiasis: *n* = 11 (18.3%).	Age range: 2–13 yr Females: *n* = 37 Males: *n* = 23	Ceftriaxone 100 mg/kg/d administered over 30 min for acute otitis media, gastroenteritis, tonsillitis, upper urinary tract infection, Kawasaki disease, phlegmon, peritonsillar abscess, cervical lymphadenitis. Fasting and bed rest were studied factors.	Pseudolithiasis occurred even in lower doses < 60 mg/kg/d. Higher risk of lithiasis was shown in patients who fasted (presented at 4–8 d) and bed rest (1–5 d).
Ozturk et al. [34]	Prospective	*n* = 33 Pseudolithiasis and gallbladder calcification: *n* = 19 (57.5%).	Mean: 76.2 ± 54.8 mo Females: *n* = 14 Males: *n* = 19	Ceftriaxone 100 mg/kg/d administered for postoperative prophylaxis (*n* = 13) and infection (*n* = 30).	Lithiasis presentation occurred between 4–8 d. Spontaneous resolution occurred within 4–21 d.
Palanduz et al. [35]	Retrospective	*n* = 118 Total gallbladder abnormalities detected: *n* = 20 (17%), including calcification (*n* = 8) and pseudolithiasis (*n* = 12).	Age range: 3 mo–14 yr Females: *n* = 66 Males: *n* = 52	Ceftriaxone 100 mg/kg/d in 2 doses over 30 min for 1–3 wk for severe infection. Study on biliary lithiasis in two groups of abnormal and normal ultrasound findings.	Detection period: 5–11 d (mean 9.1 ± 1.2 d). Resolution period: 2 wk (mean 8.2 ± 3.4 d). Age, gender, and duration of treatment were not significant factors.
Rozmanic et al. [36]	Case report	*n* = 1	Female, 9 yr	Ceftriaxone 90 mg/kg/d in a daily bolus dose for bacterial infection.	Cholelithiasis presentation after 35 h of treatment. Spontaneous resolution 2 d after treatment was discontinued.
Soysal et al. [37]	Randomized Controlled Trial	*n* = 114 At 5 d, there was biliary calcification (*n* = 14) and biliary lithiasis (*n* = 10). At 10 d, there was biliary calcification in 20 and lithiasis in 15 patients. In total, 35 patients (31%) presented biliary precipitations	Age range: 2–180 mo Mean age: 47.5 ± 46.3 mo Females: *n* = 56 Males: *n* = 58	Ceftriaxone infused over 30 min (*n* = 47), bolus over 2 min (*n* = 47) or intramuscular (*n* = 20). Mean duration of treatment: 7 d (3–14 d). Biliary ultrasonography was performed at the time of randomization before treatment was started at 5 and 10 d, and at the end of the treatment. If calcification was detected, weekly ultrasound follow-up until resolution.	Resolution occurred 10–45 d after discontinuation of treatment. Age over 12 mo, dose of more than 2 g, and duration of therapy longer than 5 d were associated with biliary precipitations.
Tuna Kirsaclioglu et al. [20]	Retrospective	*n* = 254 51 patients presented ceftriaxone-related cholelithiasis, and 7 of them presented cholecystitis.	Age range: 0.08–18 yr Mean: 8.9 ± 5.2 yr Females: *n* = 134 Males: *n* = 120	150 patients presented symptomatic and 159 asymptomatic cases of cholelithiasis of different etiologies, with ceftriaxone-related cases among them. Symptomatic patients were significantly older than the asymptomatic ones. 64 patients presented drug-related cholelithiasis.	Patients whose gallstones dissolved were significantly younger (mean age 8 ± 5.2 yr) compared to older (mean age 9.5 ± 5 yr). Ultrasound showed 40 patients under ceftriaxone with gallstones and 11 with calcification. Ceftriaxone-related cholelithiasis presented significant greater tendency for resolution compared to other etiologies. Ursodeoxycholic acid did not affect resolution time.
von Martels et al. [38]	Case Report	*n* = 1	Male, 14 yr	Abdominal pain started 4 d after discontinuation of ceftriaxone administration and continued for additional 7 d. History of ceftriaxone 4 g/d for 2 wk for Lyme arthritis.	ERCP performed Dilated intrahepatic ducts and multiple biliary precipitations in the gallbladder. After 4 mo, no gallbladder abnormalities were detected. Resolution upon ceftriaxone discontinuation.

**Table 2:** Summary of the main findings from the articles included in the study for exclusively urinary tract ceftriaxone-related complications.

Citation	Study Type	Study Patients (n)	Age, Gender	Clinical Information, Treatment	Main Outcomes
Avci et al. [39]	Prospective	*n* = 51 Nephrolithiasis: *n* = 4 (7.8%).	Age: 1 mo–14 yr Mean: 3.1 yr Females: *n* = 30 Males: *n* = 21	Ceftriaxone 100 mg/kg/d. Administered in two daily doses: *n* = 24. Administered in a single daily dose: *n* = 27. Ultrasound performed prior to and after treatment of pneumonia, pyelonephritis, lymphadenitis, meningitis, mastoiditis.	Ultrasound findings: small, echogenic calculi within dilated calices. Resolution of lithiasis occurred 3 wk after discontinuation of treatment. One patient’s stone was still present 7 mo after treatment.
Kimata et al. [17]	Retrospective	*n* = 83	Age: 3 mo–8.9 yr Mean: 30 mo Females: *n* = 43 Males: *n* = 40	Administered for bacterial pneumonia. Study in two groups: Ceftriaxone group: *n* = 43, mean dose of 91 ± 10 mg/kg/d. Amoxicillin group: *n* = 40, mean dose of 107 ± 12 mg/kg/d. Serum and urine calcium-to-creatinine ratios were measured.	Urine calcium and calcium-to-creatinine ratio were significantly higher in the ceftriaxone group. This increase occurred after ceftriaxone administration exclusively.
Lozanovski et al. [40]	Case Report	*n* = 1	Male, 5 yr	Ceftriaxone: 45 mg/kg/d. Pneumonia.	After 7 d of treatment, ceftriaxone was changed to oral cephalosporin, and there was moderate hypercalciuria without abnormal ultrasound findings. After 9 d of treatment, there were three calculi of calcium ceftriaxonate and hypercalciuria.
Mohkam et al. [41]	Prospective	*n* = 284 Ultrasound findings at 10 d: renal lithiasis in 4 patients (1.4%).	Age: 2 mo–12 yr Mean: 2.67 ± 2.10 yr Females: *n* = 185 Males: *n* = 99	Ceftriaxone: 75 mg/kg/d for at least 10 d. Pyelonephritis.	Normal ultrasound after 3 mo. Spontaneous resolution of lithiasis occurred in all patients.
Shen et al. [42]	Retrospective	*n* = 15 Multiple calculi in the upper urinary tract. Bilateral ureteral stones (*n* = 9, 60%).	Age: 5 mo–11 yr Mean: 4.76 ± 3.74 yr Females: *n* = 3 Males: *n* = 12	Ceftriaxone 1 g in one dose for 5 d. Acute kidney injury from ceftriaxone-induced urolithiasis.	No stones in ureters and kidneys 5 d after intervention (*n* = 5, 33.3%). One patient operated. All patients were followed up for 11 mo–5 yr. No recurrent urolithiasis was found.
Stojanovic et al. [19]	Care Report	*n* = 1	Male, 3 yr	Henoch Schönlein purpura complicated with pneumonia. Ceftriaxone: 100 mg/kg/d.	After 6 d of ceftriaxone administration, abdominal pain and vomit presented. Ultrasound showed calculi in the renal calyces. Immediate discontinuation of antibiotic treatment resulted in resolution of symptoms. No urinary calculi 3 wk after end of ceftriaxone administration.
Youssef et al. [22]	Prospective	*n* = 120 In the ceftriaxone group, 5 patients presented small calculi, spontaneously eliminated after 3 wk, except in one patient with renal stones after 9 mo of treatment.	Mean age: 6.93 ± 1.56 yr (ceftriaxone group) Females: *n* = 57 Males: *n* = 63	A group of 60 patients received ceftriaxone 80 mg/kg/d for 5 d and was compared to an equal number with other antibiotics. Ceftriaxone-related nephrolithiasis was studied. The 5 patients with ceftriaxone-associated nephrolithiasis had gastroenteritis (*n* = 2), meningitis, bronchopneumonia, and secondary infection after tonsillectomy.	All patients with lithiasis were asymptomatic, and urine calcium levels were normal. Mean age of nephrolithiasis (8.2 yr) was significantly higher than the group without (3.5 yr), showing older age as a possible risk factor. The study concluded that the incidence of renal stones is higher in the patients who receive ceftriaxone compared to other antibiotics.

**Table 3:** Summary of the main findings from the articles included in the study regarding combined biliary and urinary lithiasis outcomes.

Citation	Study Type	Study Patients (n)	Age, Gender	Clinical Information, Treatment	Main Outcomes
Acun et al. [43]	Case Report	One patient with ceftriaxone-induced biliary pseudolithiasis and urinary bladder calcification.	Female 5 yr	Ceftriaxone: 100 mg/kg/d in 2 doses. Infusion time over 20 min. Meningitis.	Abdominal pain after 9 d of treatment. Ultrasound: mobile gallbladder stone 1.5 mm in diameter. At 5 d, after ceftriaxone discontinuation: multiple echogenic foci in the gallbladder and calcification in the urinary bladder. At 12 d, after discontinuation: normal ultrasound findings. Urinary bladder calcification has not been reported previously.
Biner et al. [6]	Prospective	*n* = 156 *n* = 27 (17%) with bladder complications: Females: *n* = 13 Males: *n* = 14 One female patient was found with urolithiasis.	Age: 0.5–16 y, Mean: 4.5 ± 3.3 y Females: *n* = 92 Males: *n* = 64	Ceftriaxone: 100, 75, and 50 mg/kg/d for 7–10 d in short bolus injections. Pyelonephritis, pneumonia, meningitis, lymphadenitis, sepsis.	Gallstones: *n* = 16 (10.3%), calcification: *n* = 11 (7.1%), renal lithiasis: *n* = 1 (0.6%). Time of diagnosis range: 3–7 d of treatment. Complete resolution range: 10–30 d. Strong association between complications and age over 5 y, meningitis, high doses (100 mg/kg/d).
Fesharakinia et al. [44]	Prospective	*n* = 96 Persistent lithiasis after treatment: renal (*n* = 6), gallbladder (*n* = 1).	Age: 1 m–13 y Mean: 1.88 ± 1.98 y Females: *n* = 41 Males: *n* = 55	Ceftriaxone: 50–100 mg/kg/d in 2 doses in 15 min per dose. Infections included: gastroenteritis, pneumonia, septicemia, sinusitis, pyelonephritis, and febrile convulsion with acute otitis media.	Male gender significantly related to nephrolithiasis. Follow-up was defective (participants did not reappear for further examinations).
Prince et al. [45]	Case report	*n* = 1 CT scan at 8 d showed high-density material in the gallbladder, both renal pelvises and ureters.	Male, 14 yr	Ceftriaxone: 4 g/d and metronidazole. Sinusitis complicated by epidural abscess.	Immediate discontinuation of ceftriaxone and replacement by meropenem. Cystoscopy, after 9 d, revealed material in the ureters, and ureteral stents were placed. CT scan at 11 d showed decreased amount of biliary calcification and ureteral debris. CT scan after 3 wk showed complete resolution of lithiasis.
Ustyol et al. [46]	Prospective	*n* = 154 In the ceftriaxone group, 13 patients presented with biliary lithiasis, 5 with biliary calcification, and one with nephrolithiasis, while in the cefotaxime group, there were 4 patients with biliary calcification and one with nephrolithiasis.	Age: 2 mo–18 yr Mean: 4.77 ± 4.91 yr (ceftriaxone group) Females: *n* = 76 Males: *n* = 78	One group (*n* = 86) received ceftriaxone, and a second group (*n* = 68) cefotaxime. Intravenous ceftriaxone dose was 100 mg/kg/d, divided into two equal doses. Ceftriaxone administration period was 13 ± 5 d. Infections: pneumonia, pyelonephritis, lymphadenitis, bacterial meningitis, bacteraemia, gastroenteritis, peritonitis	Hypercalciuria was found in the two patients with nephrolithiasis. Abnormal ultrasound biliary findings were significantly higher in the ceftriaxone group. ROC analysis of age risk revealed that 4.5 yr was the cut-off age (sensitivity 70% and specificity 40%). According to the ROC analyses, biliary calcification development risk increased when older than 4.5 yr.

## Data Availability

Not applicable.
